# Bridging the experience gap: reasoning-oriented LLMs as cognitive scaffolds for junior anesthesiologists—a multi-arm controlled study

**DOI:** 10.1186/s12871-026-04173-4

**Published:** 2026-08-14

**Authors:** Huiju Yang, Guangyan Zhang, Zhipeng Wang

**Affiliations:** Department of Anesthesiology, Guangdong Provincial People’s Hospital (Guangdong Academy of Medical Sciences), Southern Medical University, Guangzhou, China

**Keywords:** Artificial Intelligence, Natural Language Processing, Anesthesiology, Clinical Decision-Making, Cognitive Bias, Clinical Competence

## Abstract

**Background:**

The gap between junior and senior physicians in anesthesia is exacerbated by the reliance on experience. Large language models like DeepSeek-R1 offer a potential solution as "cognitive scaffolding."

**Methods:**

A simulation-based, multi-arm controlled study with randomized junior groups and a non-randomized senior expert comparator was conducted. Forty-eight (48) anesthesiologists, including 16 senior experts and 32 junior residents, were recruited and randomly assigned (using a computer-generated randomization sequence) to two groups: Junior-alone and Junior + AI. Participants evaluated 20 standard clinical scenarios across four clinical domains: airway management, hemodynamic regulation, anesthetic pharmacology, and crisis management. The AI-assisted group followed a triple-step method: independent initial decision, subsequent AI consultation, and decision revision. Their final performance was compared against sixteen (16) senior expert anesthesiologists who served as the gold standard.

**Results:**

DeepSeek-R1 successfully improved performance in all four domains for junior doctors. Compared to the Junior-alone group, the Junior + AI group achieved significantly higher scores in accuracy (86.00 ± 16.61 vs 61.67 ± 19.45, P < 0.0001) and comprehensiveness (84.33 ± 17.18 vs 53.67 ± 23.27, P < 0.0001). However, further analysis revealed that while significantly improved, both accuracy and comprehensiveness remained lower than the senior expert scores (differences not statistically significant in mixed-effects analysis: *P = *0.349 for Accuracy; *P = *0.335 for Comprehensiveness). Performance in safety showed no significant difference to expert standards (*P = *0.644). The AI-assisted group required slightly less total time (33.3 vs 33.8 min), showing no significant delay. 65% of AI suggestions were adopted overall.

**Conclusion:**

DeepSeek-R1 shows promise as "cognitive scaffolding", supporting knowledge externalization for junior anesthesiologists. It substantially improves decision-making, though a numerical gap with experts persists in certain dimensions. The high risk of automation bias in critical scenarios requires targeted training on critical evaluation of AI outputs.

**Supplementary Information:**

The online version contains supplementary material available at https://doi.org/10.1186/s12871-026-04173-4.

## Introduction

Artificial Intelligence (AI) refers to computer programs that interact automatically and naturally with users. These programs simulate human conversation through natural language processing technology in software applications, hence the name "chatbots" [1, 2]. In recent years, the rapid development of AI technology has brought revolutionary changes to the medical field, with a significant increase in the application of AI-based chatbots in healthcare [3]. Particularly in clinical decision support systems, AI models can assist physicians in making more accurate diagnoses, selecting treatment plans, and predicting patient outcomes by analyzing vast amounts of medical data (e.g., electronic health records, medical imaging, and genomic data) [4]. With advancements in deep learning, natural language processing, and reinforcement learning, the potential of AI in clinical decision-making has become increasingly prominent, promising to improve medical efficiency, reduce human errors, and optimize patient outcomes [5].

As a discipline highly dependent on real-time decision-making, precision medication, and risk management, anesthesiology often requires clinicians to quickly access the latest guidelines, drug interaction information, or perioperative management recommendations. However, traditional clinical decision-making primarily relies on the physician's expertise and experience, while the rapid evolution of medical knowledge and the complexity of clinical data may pose challenges, leading to information overload or cognitive bias. Traditional information retrieval methods (such as consulting textbooks, medical databases, or expert consensus) may not fully meet the needs of efficient and precise clinical decision-making. Through data-driven methods, AI models can identify potential disease patterns, predict treatment responses, and provide personalized medical advice [6]. For example, in medical imaging analysis, convolutional neural networks have demonstrated diagnostic capabilities comparable to radiologists; in the field of intensive care, machine learning models can predict the risk of patient deterioration in advance, allowing for timely clinical intervention [7]. Given these advances, AI language models with powerful natural language processing capabilities and knowledge integration potential are expected to become powerful tools for anesthesiologists [8].

Although AI language models perform excellently in medical question-answering tasks and can provide conversational and seemingly authoritative responses to complex medical questions, they may also generate biased or incorrect answers. Therefore, caution must be maintained when considering their application in medical practice and research [9–13]. Furthermore, while traditional Large Language Models (LLMs) have demonstrated exceptional capabilities in knowledge retrieval, their operational mode often resembles a "System 1" thinking engine—capable of providing fast, intuitive responses but lacking the rigorous multi-step reasoning required for high-risk perioperative management [14]. The emergence of reasoning-oriented models, represented by DeepSeek-R1, marks a paradigm shift. Through large-scale reinforcement learning and explicit Chain of Thought (CoT) processing mechanisms, they internalize the "System 2" thinking mode as a core capability [15, 16]. This characteristic is particularly critical in the field of anesthesiology because clinical decisions require the simultaneous integration of multiple interdependent physiological systems [14]. However, the extent to which these reasoning capabilities can bridge the "experience gap" between senior and junior physicians while avoiding common clinical pitfalls remains to be explored in depth [17].

Therefore, this study aims to evaluate the protective effect of reasoning models, represented by DeepSeek-R1, on the clinical safety boundaries of junior physicians in terms of accuracy, comprehensiveness, and safety when answering anesthesia-related questions, revealing its potential and limitations in handling the complex and ambiguous situations faced by doctors and patients in clinical practice.

## Materials and methods

### Research design

This simulation-based, multi-arm controlled study with randomized junior groups and a non-randomized senior expert comparator was conducted at Guangdong Provincial People's Hospital from September 2025 to March 2026. The research protocol was approved by the Ethics Committee of Guangdong Provincial People's Hospital (Approval No.: KY-Z-2021–629-04) and followed the Declaration of Helsinki. All participants signed written informed consent.

Reporting follows the CONSORT-AI (Consolidated Standards of Reporting Trials-Artificial Intelligence) guidelines [18]. The AI intervention is described in detail: Model: DeepSeek-R1 (DeepSeek AI, Hangzhou, China), accessed via the official web interface (https://www.deepseek.com/). Input: Standardized clinical scenario text entered by participants using a uniform prompt template. Output: Free-text reasoning and recommendations generated by the model. Human-AI interaction: Three-step protocol (independent decision, AI consultation, decision revision), with participants free to adopt, modify, or reject AI suggestions. Error handling: Five scenarios contained investigator-designed simulated erroneous AI advice. User setting: Junior anesthesiologists (1–3 years experience) in a simulated examination environment. Workflow context: Offline clinical decision-making simulation, not integrated into real-time clinical workflow.

### Participants

A total of thirty-two (32) junior anesthesiologists (1–3 years of clinical training in anesthesia) were recruited from Guangdong Provincial People's Hospital. Eligible participants were stratified by year of training and then randomized into two parallel groups (1:1 ratio) using a computer-generated randomization sequence (BlockRandomization V1.0), which ensured that the Junior physicians (n = 16) and Junior physicians with the assistance of the DeepSeek-R1 model (n = 16) groups were comparable in baseline clinical experience and training (Table 1, P > 0.05 for all baseline characteristics). Allocation concealment was maintained using sequentially numbered, opaque, sealed envelopes. Additionally, 16 senior anesthesiologists (> 15 years of post-training experience) were recruited to serve as the senior expert benchmark.

**Table 1:** Baseline characteristics of Junior-alone and Junior + AI groups (randomization balance check)

Variable	J-alone group (n = 16)	J + AI group (n = 16)	*P*-value
Age (yr)	32 ± 3.87	29.81 ± 3.49	0.08
Mean years of experience	3.06 ± 0.85	2.94 ± 0.71	0.66
Independent cases	35.06 ± 15.69	35.75 ± 13.04	0.91
Professional title			
Residents	16	16	-
Prior AI usage	0	0	-

Note 1: Values are presented as mean ± SD or numbers. All P > 0.05, confirming successful randomization balance between the two junior groups. J-alone: Junior-alone; J + AI: Junior + AI

Participants included 16 senior physicians:

Inclusion criteria:

1. Clinical experience: ≥ 15 years of practice as an attending physician or above.
2. Independent decision-making ability: Completed ≥ 200 cases of anesthesia as the primary anesthesiologist, including complex procedures.

Exclusion criteria:

1. Did not participate in frontline clinical work in the past year.
2. Frequent prior use of AI-assisted anesthesia tools.

And 32 junior physicians:

Inclusion criteria:

1. Clinical experience: ≤ 3 years of practice as a resident.
2. Independent decision-making ability: Have not served as the primary anesthesiologist and have completed ≤ 50 cases of general anesthesia independently.

Exclusion criteria:

1. Previously participated in similar AI-assisted anesthesia research.
2. Received advanced anesthesia decision-making training within the past 6 months.

Sample size adequacy was verified through power analysis using G*Power software (effect size f^2^ [V] = 0.15, α = 0.05, total sample size = 48, actual power = 84%). Participants were divided into three groups (n = 16 per group): (1) Senior group: Senior physicians answered independently. (2) Junior-alone group: Junior physicians answered independently. (3) Junior + AI group: Junior physicians answered with the assistance of the DeepSeek-R1 model.

To minimize potential confounding factors, baseline characteristics were collected, including age and the number of anesthesia cases handled in the past year. Additionally, to ensure environmental consistency, all participants accessed the DeepSeek-R1 model through a standardized interface that restricted access to external medical databases, ensuring that all feedback reflected only the interaction between the physician's judgment and the model's reasoning. To quantify the net gain of DeepSeek-R1 on clinical thinking, a three-step "Independent Decision – AI Consultation – Decision Revision" method was adopted: All junior and senior participants reviewed the same 20 cases.

This restriction was implemented for two reasons: (1) to ensure experimental control and internal validity—all participants received AI assistance from the same model with identical knowledge parameters, eliminating confounding from variable external database access; and (2) to specifically evaluate the reasoning capability of DeepSeek-R1 itself, rather than its ability to retrieve external information. The core research question pertained to whether the model's internalized reasoning (System 2 thinking) could serve as a cognitive scaffold.

Senior group: Formulated independent plans, which served as the gold standard.

Junior-alone group: Formulated independent plans without external assistance.

Junior + AI group: Followed a structured, three-step consultation model to precisely quantify the AI's "net effect" on their decisions:

1. Independent Human Decision: For each case, the junior doctor first formulated and documented their initial, independent anesthesia and anesthetic pharmacologyplan, establishing a baseline.
2. AI Consultation: After submitting their initial plan, participants received complete access to the specific analysis and suggestions generated by J-DeepSeek-R1 for that same case. For the implanted simulated AI error cases, the AI’s erroneous advice was presented to the participants as generated, without pre-filtering.
3. Decision Revision: Participants reviewed their initial plan alongside the AI's input and could choose to adopt, adapt, or reject the AI's suggestions to produce a final, definitive decision. The score of this final decision (not the baseline initial decision) was used for comparison with the other groups.

### Testing process

All participants were required to complete a test consisting of 20 anesthesia-related clinical questions. Although the total sample size consisted of 48 participants, each physician independently evaluated 20 meticulously designed clinical scenarios. This multi-dimensional testing approach resulted in a robust dataset of 960 decision-making records for analysis. These observations are nested within participants and scenarios; accordingly, all primary analyses employ mixed-effects models with random effects for participant and scenario to appropriately handle this clustered data structure (see Statistical Analysis). These questions were developed by an expert panel using the Delphi method based on real clinical scenarios, designed to reflect the daily decision-making challenges of anesthesiologists, covering core competencies such as airway management, hemodynamic regulation, anesthetic pharmacology, and crisis management, and aligned with clinical guideline consensus standards published in Anesthesiology journals. To further evaluate the model's performance in different clinical domains, the expert panel pre-classified the 20 questions into four subcategories: (1) Airway management (focusing on anatomical assessment and operational intuition); (2) Hemodynamic regulation (focusing on physiological logic and multi-step reasoning); (3) Anesthetic pharmacology (focusing on dose calculation and drug interactions); (4) Crisis management (focusing on emergency protocols), with 5 questions in each category to ensure a balanced comparison.

Twenty (20) anesthesia cases were developed to represent complex clinical scenarios across four domains: airway management, hemodynamic regulation, anesthetic pharmacology, and crisis management. Each case required participants to formulate a complete plan, which was scored across four dimensions: Accuracy, Comprehensiveness, Safety, and Response Time. To test the risk of automation bias, five specific scenarios were designed to embed subtle, plausible AI hallucinations (e.g., misidentified anatomical features leading to an inappropriate induction technique, and a misdiagnosed cause of hypotension).

To assess automation bias and clinical safety boundaries, we designed simulated erroneous AI advice and embedded them within the AI consultation responses in 25% of the test cases. these errors were evenly distributed among the subgroups, including: 2 dosage errors (e.g., mistaking μg for mg in the pharmacology group), 2 serious contraindication omissions (e.g., ignoring a patient's severe valvular stenosis in the circulatory management group), and 1 drug time-effect deviation (e.g., incorrectly describing the onset and metabolism time of muscle relaxants in the airway management group). These errors were designed to a degree that senior physicians could easily detect, but junior physicians who were fatigued or over-reliant on AI might overlook, aiming to stress-test the "cognitive defense" capabilities of the physicians. The Senior group and Junior-alone group answered independently, while the Junior + AI group was required to enter the following prompt into the DeepSeek platform: "Please provide a detailed answer to the following anesthesia-related question (specific content) from a clinical perspective. The answer should have clear logic and be presented in bullet points." All participants completed the answers simultaneously in three independent designated rooms, and each question had to be answered in a new conversation thread. When using AI assistance, participants were required to record the time spent in the electronic medical record system. By comparing system logs with the final answers, we analyzed whether the model's suggestions were modified or supplemented, and then calculated the adoption rate and modification rate (adoption refers to the junior physician integrating the AI-generated answer based on their own knowledge; modification refers to adjusting specific details, such as adjusting anesthesia doses for different patients, rather than direct copying).

It should be noted that these errors were not naturally generated by DeepSeek-R1 but were investigator-designed and manually inserted into the AI response templates to simulate plausible AI hallucination scenarios.

### Clinical reasoning assessment

Two senior experts, who did not participate in answering or scoring, performed double-blind scoring (unaware of the grouping and participant information). Each response was evaluated using a 5-point Likert scale across three dimensions: (1) Accuracy: 0 (completely incorrect) to 5 (completely correct with sound reasoning), total 100 points. (2) Comprehensiveness: 0 (missing key elements) to 5 (including full procedural details), total 100 points. (3) Safety: 0 (dangerous) to 5 (safe), total 100 points. Each of the 20 scenarios was scored 0–5, yielding a maximum of 100 points per dimension.

In addition to providing these three overall scores, the two double-blind (To maintain blinding, all responses were converted into plain text format by a research assistant before being presented to the evaluators, ensuring the AI's structured formatting did not reveal the group allocation.) experts also calculated scores for the four sub-dimensions: airway management, hemodynamic regulation, anesthetic pharmacology, and crisis management:

$$Dimension\:Score=\frac{Sum\,of\,scores\,for\,all\,questions\,in\,that\,dimesion}{Number\,of\,questions\,in\,that\,dimension}$$

The Experience Gap Reduction Index (EGRI) for each dimension (quantifying the effectiveness of AI in bridging the performance difference between junior and senior physicians) was calculated as follows:

The EGRI is a novel metric developed by the authors for this study to quantify the effectiveness of AI in bridging the performance difference between junior and senior physicians. It has not been previously validated in other contexts. An EGRI of 100% indicates complete gap closure, while 0% indicates no improvement. EGRI = [(Junior + AI score—Junior-alone score)/(Senior score—Junior-alone score)] × 100%.

$$EGRI=\frac{\left(Junior+AI score\right)-\left(Junior-alone score\right)}{Senior score-Junior-alone score}\times 100$$

Adoption and modification rates (expressed as percentages) were calculated based on all responses and tracked using system logs. The final score was the average of the two raters. The time taken by each participant to answer was recorded.

### Statistical analysis

Data are expressed as mean ± standard deviation. To account for the clustered data structure (20 scenarios nested within each participant), linear mixed-effects models (LMMs) were used as the primary analysis, with fixed effects for group (Senior, Junior-alone, Junior + AI) and random intercepts for participant and scenario. This approach appropriately handles the non-independence of repeated observations within each physician and each case. Pairwise comparisons between groups were performed using estimated marginal means with Tukey adjustment for multiple comparisons. The senior expert group served as the reference category. As a sensitivity analysis, participant-level summary statistics (mean score per participant across all 20 scenarios) were also analyzed using one-way ANOVA with Dunnett's post-hoc test (using the Senior group as reference) to confirm the robustness of the mixed-effects results. Homogeneity of variance was confirmed by Bartlett's test (p > 0.05). Inter-rater reliability was assessed using the intraclass correlation coefficient (ICC) and Cohen's Kappa. For the subgroup analyses across four clinical domains, mixed-effects models were similarly applied with the domain included as an additional fixed effect. The Experience Gap Reduction Index (EGRI) was calculated per domain. Analysis was performed using R version 4.3.0 (lme4 and emmeans packages) and SPSS 22.0.

**Fig. 1:**
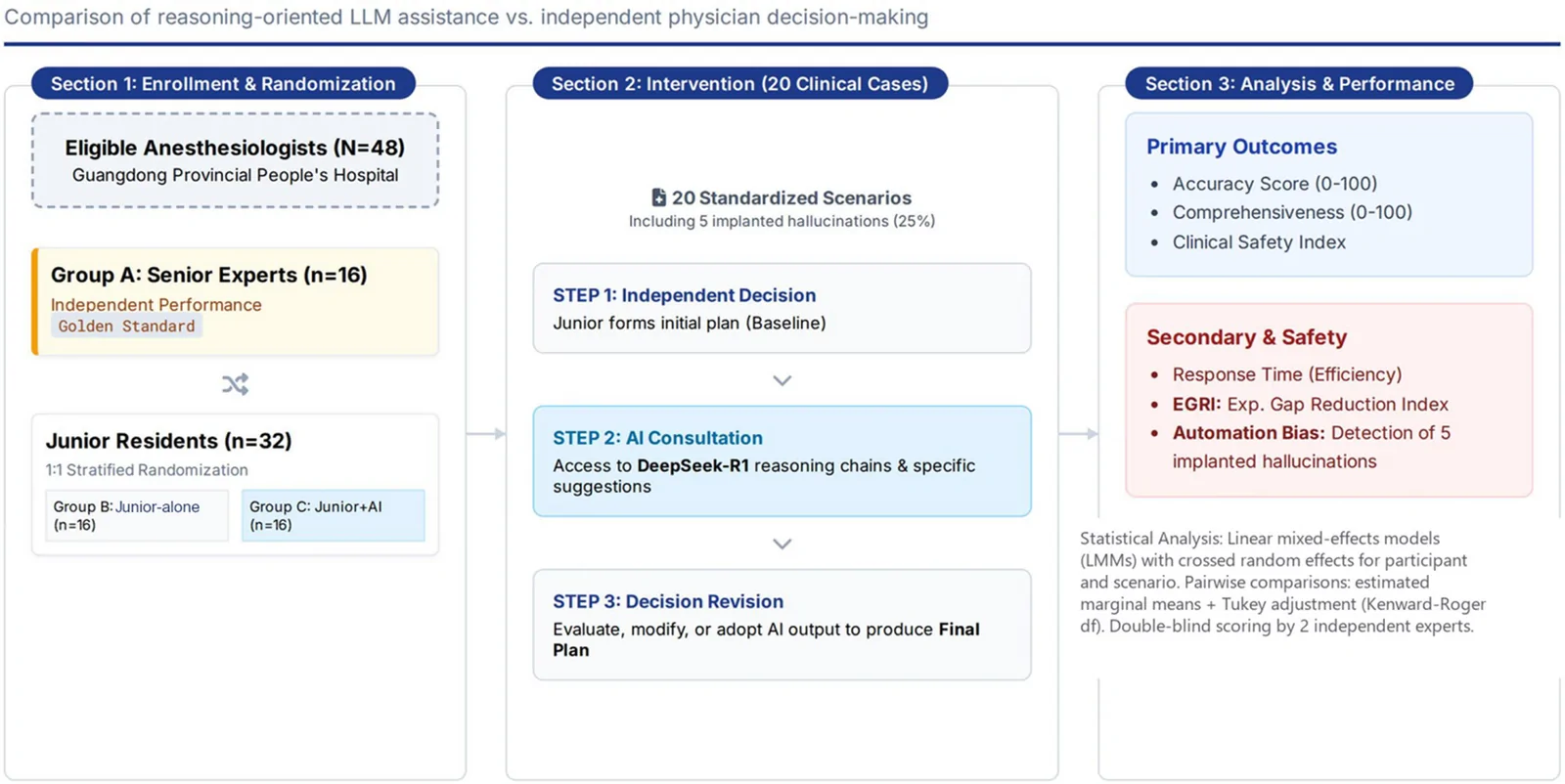
Flow chart of the research

The specific process is shown in Fig. 1.

## Results

A total of 48 physicians were included in this study, including 16 senior physicians (mean years of experience: 15 years) and 32 junior physicians (mean years of experience: 3 years). Baseline data are shown in Supplementary Table S1. The free, publicly available version of DeepSeek-R1 was used (https://www.deepseek.com/), accessed via web browser. No paid API or premium subscription was utilized. The inter-rater reliability intraclass correlation coefficient was 0.89, and the Kappa coefficient was 0.76. The Junior-alone and Junior + AI groups were comparable at baseline (Table 1, all P > 0.05), confirming successful randomization.

The scores of the three groups in the dimensions of accuracy, comprehensiveness, and safety, as well as the average response time, are shown in Table 2. Specifically: A total of 960 decision-making records (48 participants × 20 scenarios) were analyzed using linear mixed-effects models with random intercepts for participant and scenario to account for the clustered data structure. The intraclass correlation coefficient (ICC) for participant was 0.404, 0.402, and 0.444 for accuracy, comprehensiveness, and safety respectively, confirming substantial within-participant correlation and supporting the use of mixed-effects models over simple ANOVA. See Table 3 for full LMM pairwise comparison details.

**Table 2:** Comparison of scores in Accuracy, Comprehensiveness, Safety and response time among the three groups

Dimension	J-alone group	J + AI group	Senior group
Accuracy	61.67 ± 19.45^****^	86.00 ± 16.61^ ns^	91.67 ± 15.07
Comprehensiveness	53.67 ± 23.27^****^	84.33 ± 17.18^ ns^	90.67 ± 15.19
Safety	70.67 ± 19.88^****^	90.33 ± 18.46^ns^	94.33 ± 17.43
Average response time	33.8	33.3	28.1

Note 2: Values are presented as mean ± SD. Response time is presented in minutes (min). Comparisons were performed using linear mixed-effects models (LMMs) with fixed effects for group and random intercepts for participant and scenario, followed by pairwise comparisons using estimated marginal means with Tukey HSD adjustment. The Senior group served as the reference category. As a sensitivity analysis, participant-level summary means were also compared using one-way ANOVA with Dunnett's post-hoc test, yielding consistent results (Supplementary Table S2). Significance symbols refer to pairwise comparisons against the Senior group. ns, P > 0.05; **** *P* < 0.0001. In addition, J + AI vs J-alone was highly significant for all three dimensions (Accuracy: P < 0.0001; Comprehensiveness: P < 0.0001; Safety: *P = *0.0002). See Table 3 for full LMM pairwise comparison details. ICC(participant) ranged from 0.40 to 0.44, confirming the clustered data structure. J-alone: Junior-alone; J + AI: Junior + AI

**Table 3:** Linear mixed-effects model pairwise comparisons and variance components

Dimension	Comparison	Estimate	SE	df	t ratio	95% CI	P value	Sig
Accuracy	Senior vs J + AI	5.67	4.04	45	1.40	[−4.13, 15.47]	0.349	ns
Accuracy	Senior vs J-alone	30.00	4.04	45	7.42	[20.20, 39.80]	< 0.0001	****
Accuracy	J + AI vs J-alone	24.33	4.04	45	6.02	[14.53, 34.13]	< 0.0001	****
Comprehensiveness	Senior vs J + AI	6.34	4.44	45	1.43	[−4.41, 17.09]	0.335	ns
Comprehensiveness	Senior vs J-alone	37.00	4.44	45	8.34	[26.25, 47.75]	< 0.0001	****
Comprehensiveness	J + AI vs J-alone	30.66	4.44	45	6.91	[19.91, 41.41]	< 0.0001	****
Safety	Senior vs J + AI	4.00	4.45	45	0.90	[−6.79, 14.79]	0.644	ns
Safety	Senior vs J-alone	23.66	4.45	45	5.31	[12.87, 34.45]	< 0.0001	****
Safety	J + AI vs J-alone	19.66	4.45	45	4.42	[8.87, 30.45]	0.0002	***

Note3: LMMs were fitted with lmer(score ~ group + (1|participant) + (1|scenario), REML = TRUE). Pairwise comparisons used estimated marginal means with Tukey HSD adjustment. Degrees of freedom estimated using the Kenward-Roger method. The senior expert group served as the reference category

### Accuracy

**Fig. 2:**
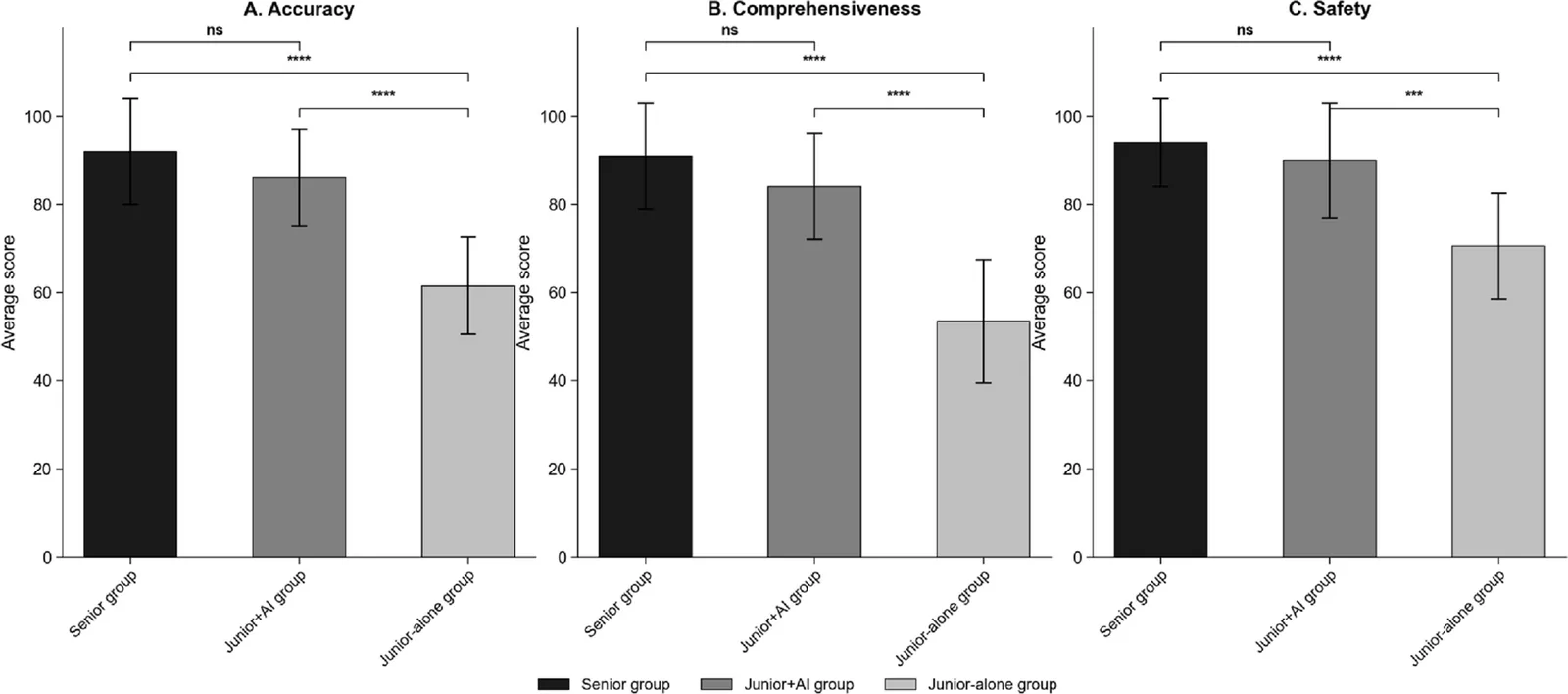
Quantitative assessment of Senior, Junior-alone and Junior + AI groups across three core clinical dimensions(Mean ± SD)

The Senior group scored the highest (91.67 ± 15.07), followed by the Junior + AI group (86.00 ± 16.61), and the Junior-alone group was the lowest (61.67 ± 19.45) (Fig. 2A). In the linear mixed-effects model, the Junior + AI group approached the Senior expert level, though the difference did not reach statistical significance (Estimate = 5.67, 95% CI [−4.13, 15.47], Tukey-adjusted *P = *0.349, ns). In contrast, the Junior-alone group scored significantly lower than both the Senior group (*P* < 0.0001) and the Junior + AI group (*P* < 0.0001), confirming both the existence of an experience gap and the significant improvement conferred by AI assistance (Table 4).

**Table 4:** Variance components and intraclass correlation coefficients (ICC)

Dimension	sigma^2 (participant)	sigma^2 (scenario)	sigma^2 (residual)	ICC (participant)	ICC (scenario)
Accuracy	122.23	8.19	171.97	0.404	0.027
Comprehensiveness	146.97	9.65	209.47	0.402	0.026
Safety	149.39	2.55	184.82	0.444	0.008

ICC(participant) = sigma^2(participant)/[sigma^2(participant) + sigma^2(scenario) + sigma^2(residual)]

### Comprehensiveness

The Senior group remained in the lead (90.67 ± 15.19), followed by the Junior + AI group (84.33 ± 17.18), and the Junior-alone group was the lowest (53.67 ± 23.27) (Fig. 2B). In the linear mixed-effects model, the Junior + AI group approached the Senior expert level, with the difference not reaching statistical significance (Estimate = 6.34, 95% CI [−4.41, 17.09], Tukey-adjusted *P = *0.335, ns). The Junior-alone group scored significantly lower than both the Senior group (*P* < 0.0001) and the Junior + AI group (*P* < 0.0001).

The sensitivity analysis using participant-level summary statistics (one-way ANOVA with Dunnett's post-hoc test) confirmed these results: Senior vs Junior + AI for accuracy (*P = *0.374, ns) and comprehensiveness (*P = *0.359, ns). See Supplementary Table S2 for full sensitivity analysis details including Cohen's d effect sizes.

### Safety

There was no statistically significant difference between the scores of the Senior group (94.33 ± 17.43) and the Junior + AI group (90.33 ± 18.46) (p > 0.05, ns). Both groups were significantly higher than the independent Junior-alone group (70.67 ± 19.88) (Fig. 2C). This result suggests that with the assistance of DeepSeek-R1, the clinical safety decision-making ability of junior physicians reached a level comparable to that of senior physicians. In the linear mixed-effects model, the difference between the Senior and Junior + AI groups was not statistically significant (Estimate = 4.00, 95% CI [−6.79, 14.79], Tukey-adjusted *P = *0.644, ns). The Junior-alone group scored significantly lower than both the Senior group (*P* < 0.0001) and the Junior + AI group (*P = *0.0002).

**Fig. 3:**
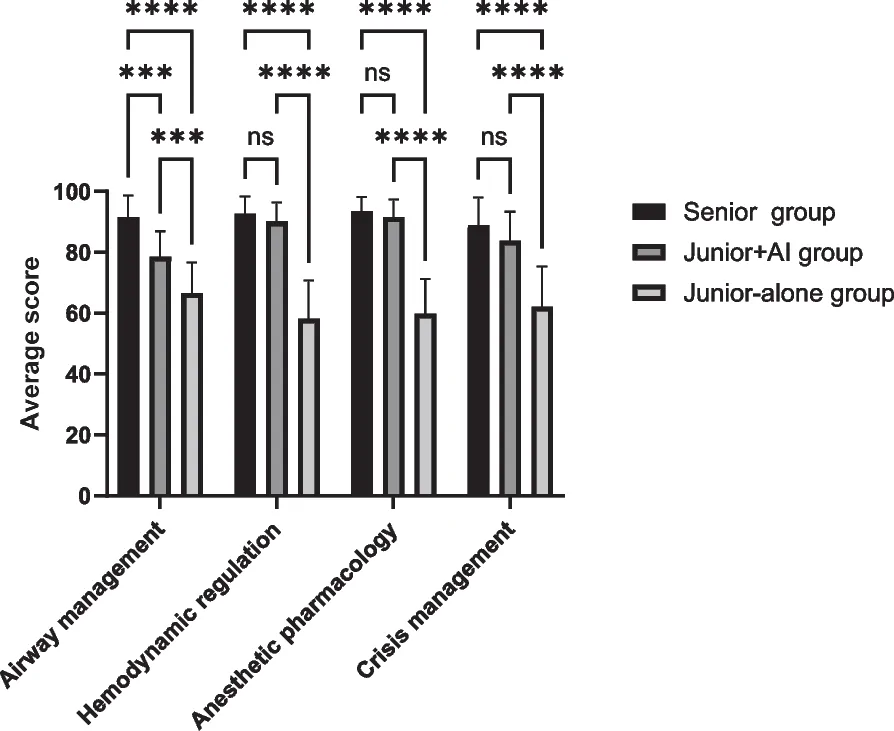
Comparison of clinical reasoning performance across four specialized anesthesia dimensions among Senior, Junior-alone and Junior + AI groups

Subgroup analysis showed (Table 5) that the clinical improvement brought by DeepSeek-R1 varied across different fields. In the areas of hemodynamic regulation and anesthetic pharmacology, the scores obtained by the Junior + AI group (90.24 ± 6.13 and 91.52 ± 5.83, respectively) showed no statistical difference compared to the Senior group (92.82 ± 5.52 and 93.51 ± 4.82) (p > 0.05). However, in the field of airway management, although AI assistance improved the scores of junior physicians, a significant gap remained between the Junior + AI group and Senior group (78.53 ± 8.44 vs 91.54 ± 7.24, *P* < 0.001) (Fig. 3). The EGRI for the four dimensions were Airway management (48%), Hemodynamic regulation (92%), Anesthetic pharmacology (94%), and Crisis management (81%).

**Table 5:** Performance comparison and EGRI across Anesthesia subspecialties

Clinical Subgroups	J-alone group	J + AI group	Senior group	EGRI
Airway management	66.51 ± 10.22^****^	78.53 ± 8.44^***^	91.54 ± 7.24	48%
Hemodynamic regulation	58.21 ± 12.53^****^	90.24 ± 6.13^ ns^	92.82 ± 5.52	92%
Anesthetic pharmacology	59.82 ± 11.44^****^	91.52 ± 5.83^ ns^	93.51 ± 4.82	94%
Crisis management	62.24 ± 13.12^****^	83.84 ± 9.53^ ns^	88.91 ± 9.24	81%

Note 4: Values are presented as mean ± SD or numbers. EGRI is presented as numbers (%). EGRI: Experience Gap Reduction Index. Comparisons were made with the Senior physician group using Dunnett’s post-hoc test. ns, *P* > 0.05. ^***^ *P* < 0.001. ^****^ *P* < 0.0001. J-alone: Junior-alone; J + AI: Junior + AI

In the error typology analysis, we adopted a simulated AI error taxonomy. Among them, the detection rate for dosage errors was the highest (34.38%), while the detection rates for more logically concealed contraindication omissions and time-effect deviations were only 28.13% and 12.5%, respectively, showing obvious automation bias. See Table 6 for details.

**Table 6:** Physician identification and response to simulated erroneous AI advice

Error types	Total AI errors	Junior detection rate(%) (detected/total)	Senior detection rate(%) (detected/total)
Dosage errors	2	34.38% (11/32)	93.75%(30/32)
Contraindication omissions	2	28.13% (9/32)	90.63%(29/32)
Time-effect deviations	1	12.5% (2/16)	87.5%(14/16)

Note 5: Detection rate = (number of times the error was identified)/(number of junior physicians x number of scenarios containing that error type). Each physician independently evaluated each scenario

After calculation, the actual clinical adoption rate of suggestions provided by the DeepSeek-R1 model was 65%, and the actual clinical modification rate was 35%.

## Discussion

This study compared how three groups—Senior, Junior-alone, and Junior + AI—performed on 20 realistic anesthesia questions. We assessed accuracy, comprehensiveness, safety, response time, and their handling of specific errors. Results showed that DeepSeek-R1 enhanced answer quality, suggesting it can help structure clinical reasoning and support junior physicians in complex scenarios, though real-world validation is still needed.

The training cycle for anesthesiologists is long. Research shows that it takes an average of over 10 years for junior physicians to reach the level of senior physicians [19]. The shortage of senior physicians is becoming increasingly severe globally. In this context, AI-assisted decision-making can enhance the clinical diagnosis and treatment efficiency of junior physicians. For example: 1. AI can integrate more evidence-based medical evidence, improving the comprehensiveness of decisions and assisting in the clinical assessment of complex cases [20]; 2. AI can also assist surgeons in intraoperative decision-making [21].

DeepSeek-R1 excels in logical reasoning within scientific fields, enhancing mathematical and complex problem-solving abilities. However, anesthesia decision-making combines both "science" (precision medication, physiological regulation) and "humanities" (communication, ethical considerations). This study found that R1 achieves a good balance between the two. These results highlight the inherent "System 2" reasoning characteristics of DeepSeek-R1. Its strengths in circulation and drug management derive from processing complex multi-step logic chains and precise physiological calculations—highly structured tasks. In contrast, its more limited improvement in airway management suggests that "clinical intuition"—gained from high-risk physical operations and rapid pattern recognition—remains a domain where senior anesthesiologists hold a significant advantage. Current text-based reasoning models cannot yet fully replicate this core clinical competency. Compared to similar studies (such as GPT-4's performance in anesthesia Q&A) [14], the DeepSeek-R1 model has advantages in the Chinese medical context, especially regarding the accuracy of professional terminology. However, it must be noted that AI can still make mistakes, and the responsibility remains with the physician; therefore, physicians must maintain critical thinking. Despite the aforementioned performance improvements, we hold a cautious attitude toward the finding of a 35% modification rate. While some clinicians corrected AI errors by exercising "professional discretion," the high adoption rate of 65% may suggest a potential risk of "automation bias," where junior physicians may experience "algorithmic trust overload" when faced with highly logical AI suggestions. In the 35% of modification cases, physicians mostly focused on individual micro-adjustments of anesthesia doses (based on weight or comorbidities), while the detection rate for logical "simulated AI errors" remained low. This suggests that future AI assistance tools should mandatorily introduce a "critical proofreading" phase rather than merely providing final answers. Recent trials have shown that even physicians trained in AI can be misled by incorrect suggestions presented in the fluent narrative style unique to reasoning models. This risk is exacerbated by the "cognitive offloading" phenomenon—when provided with highly professional solutions generated by AI, clinicians subconsciously reduce their own cognitive input [22]. Without rigorous human supervision, over-reliance on such tools could lead to "clinical skill degradation" or "cognitive atrophy" in the long run [23]. Given the dynamic, time-critical, and highly hands-on nature of anesthesiology, our findings strongly suggest that current reasoning LLMs are not yet suitable for real-time intraoperative emergency guidance. Instead, their clinical translation should be strictly confined to "cold scenarios." Specifically, these models demonstrate immediate translational value in preoperative complex case planning, postoperative anesthetic pharmacology reviews, and as offline cognitive simulation training tools for residents. To bridge the gap to clinical integration, we propose developing an "asynchronous trigger mechanism" within the Electronic Medical Record (EMR) system. For instance, when a junior physician prescribes a complex postoperative patient-controlled analgesia (PCA) regimen, the AI could run a background logical validation and generate alert pop-ups for potential contraindications or dosage errors, thereby embedding the tool into the real-world clinical workflow without interrupting real-time patient care.

The Chain of Thought (CoT) mechanism of DeepSeek-R1 highly aligns with the clinical logic of anesthesiologists. For example, its multi-step reasoning in handling complex circulatory fluctuations—from ruling out hypovolemia to evaluating myocardial contractility—provides essential cognitive pathway support for junior physicians. Notably, AI assistance not only improved accuracy but also eliminated the statistical safety difference between junior and senior groups, demonstrating the potential of reasoning AI as a "safety firewall" in high-risk medical decisions. Through error typology analysis, this study identified potential risk patterns in the model's assistance, such as dosage errors, contraindication omissions, and time-effect deviations. These findings suggest that when using AI systems, a real-time contraindication check module should be embedded, and training on dose calculation and contraindication verification should be strengthened. Furthermore, an error pattern feedback loop should be established to provide real-time clinical feedback for model iteration, and specialized verification plugins should be developed to constrain the model's output range. The discovered "equalizing effect" holds profound public health implications for regions with scarce medical resources and limited senior physicians. Ultimately, DeepSeek-R1 does not replace doctors but reshapes them, transforming junior physicians into "augmented physicians" equipped with expert-level decision support.

Although this study confirms that DeepSeek-R1 possesses certain potential in clinical anesthesia decision-making, limitations remain. 1. As a proof-of-concept pilot study, the sample size of 48 participants is relatively small, although the generation of 960 decision nodes partially mitigates this limitation. 2. Only one AI model (DeepSeek-R1) was evaluated; performance may differ with other models or versions; 3. The examination-based design may not fully capture the cognitive demands of real-time clinical decision-making under time pressure and patient interaction; 4. It lacks cross-cultural validation. To address these shortcomings and further evaluate the true clinical impact and safety boundaries, future research must involve multi-center, large-sample randomized controlled trials (RCTs) and include more AI models from different brands (e.g., GPT-5, ERNIE Bot, Claude).

5. The simulated error scenarios were investigator-designed rather than naturally occurring AI hallucinations, limiting generalizability to real-world AI error patterns. 6. The EGRI is a newly proposed metric that lacks external validation. 7. The study was conducted at a single center with a homogeneous participant population, limiting generalizability.

## Conclusions

In summary, DeepSeek-R1 acts not merely as an answer provider but as a "cognitive support tool" that enhances the clinical reasoning of junior anesthesiologists. To ensure patient safety, its application must adhere to the principle of "augmentation, not replacement." We recommend that future residency training shift its focus toward cultivating "AI literacy" and "collaborative prudence," thereby equipping the next generation of physicians to lead the AI transformation in healthcare.

## Supplementary Information

Supplementary Materia 1.

## Data Availability

The datasets used and/or analysed during the current study are available from the corresponding author on reasonable request.
