# Supplementary material for: Bridging the experience gap: reasoning-oriented LLMs as cognitive scaffolds for junior anesthesiologists—a multi-arm controlled study
Source: BMC Anesthesiol. 2026 Aug 14;26:669. doi: 10.1186/s12871-026-04173-4 (PMC13749728; doi:10.1186/s12871-026-04173-4)
Supplement: Supplementary file 1 [file 12871_2026_4173_MOESM1_ESM.docx]

Supplementary Material

Supplementary Table S1. Baseline characteristics of all participating anesthesiologists (Senior vs Total Junior)

| Variable | Senior group（n=16） | Total Junior group（n=32） | P-value |
| --- | --- | --- | --- |
| Age (yr) | 45$\pm$12.28 | 30$.91\pm3.84$ | <0.05 |
| Mean years of experience | 15 | 3 | <0.05 |
| Independent cases | ≥200 | ≤50 | <0.05 |
| Professional title |  |  |  |
| Attending or above | 16 | 0 | <0.05 |
| Residents | 0 | 32 | <0.05 |
| Prior AI usage | 0 | 0 | - |

Note: Values are presented as mean ± SD or numbers.

Supplementary Table S2. Sensitivity analysis: participant-level summary statistics with one-way ANOVA and Dunnett's post-hoc test, including Cohen's d effect sizes

| Dimension | Comparison | Mean diff | t ratio | P (Dunnett) | Sig | Cohen's d |
| --- | --- | --- | --- | --- | --- | --- |
| Accuracy | Senior vs J+AI | 5.67 | 1.40 | 0.374 | ns | -0.50 |
| Accuracy | Senior vs J-alone | 30.00 | 7.42 | <0.0001 | **** | -2.61 |
| Accuracy | J+AI vs J-alone | 24.33 | 6.02 | <0.0001 | **** | 2.12 |
| Comprehensiveness | Senior vs J+AI | 6.34 | 1.43 | 0.359 | ns | -0.54 |
| Comprehensiveness | Senior vs J-alone | 37.00 | 8.34 | <0.0001 | **** | -2.90 |
| Comprehensiveness | J+AI vs J-alone | 30.66 | 6.91 | <0.0001 | **** | 2.35 |
| Safety | Senior vs J+AI | 4.00 | 0.90 | 0.681 | ns | -0.31 |
| Safety | Senior vs J-alone | 23.66 | 5.31 | <0.0001 | **** | -1.93 |
| Safety | J+AI vs J-alone | 19.66 | 4.42 | 0.0002 | *** | 1.59 |

Note: Each participant's mean score across 20 scenarios served as a single observation (n=16 per group). One-way ANOVA with Dunnett's post-hoc test (Senior as reference) was used. Cohen's d was calculated as (mean1 - mean2) / pooled SD. ANOVA F-statistics: Accuracy F=31.07 (P<0.0001); Comprehensiveness F=39.79 (P<0.0001); Safety F=16.18 (P<0.0001). All results are consistent with the LMM primary analysis (Table 3), confirming the robustness of the findings. J-alone: Junior-alone; J+AI: Junior+AI.
